# Local-scale increase masks landscape-scale loss of species richness in managed Pannonian grasslands

**DOI:** 10.1007/s10980-025-02256-0

**Published:** 2025-11-25

**Authors:** Elias Kapitany, Thomas Wrbka, Stefan Dullinger

**Affiliations:** Division of Biodiversity Dynamics and Conservation, Department of Botany and Biodiversity Research, University of Vienna, Wien, Austria

**Keywords:** Grasslands, Biodiversity, Vegetation, Landscape, Resurvey, Species trends

## Abstract

**Context:**

While biodiversity decline is undebated on the global level, landscape-scale trends are poorly known and local assemblages even show stable species richness, accompanied by pronounced turn-over. The landscape-scale consequences of local-scale species turnover likely depend on whether species replacement is random or biased towards more frequent species in the metacommunity, but this potential bias is insufficiently studied.

**Objectives:**

Here, we use grassland ecosystems of a Central European national park to simultaneously analyse time-series of local-scale species richness and landscape-scale species incidence to better understand how trends are linked at these two scales.

**Methods:**

From 2013 to 2024 we sampled 120 plots per year and used regression methods to quantify changes in the number of species per assemblage, the incidence of species across assemblages and the relationship between initial incidence of species and incidence trends. To explore possible drivers of change, we further evaluated trends of community means of environmental indicator values.

**Results:**

We found that local species richness has increased within the study period from 18 species per plot in 2013 to 21 species in 2024, while the overall number of species sampled per year stayed the same. In contrast, when looking at individual species trends we found an average decline of species’ incidence in the region. While a small pool of already common species became more frequent, the majority of species became rarer, leading to a pronounced homogenization of plant communities on the sampled sites. Indicator-value analysis showed that the species turnover was mainly influenced by desiccation of grasslands, significantly biassing incidence changes towards species that prefer drier conditions.

**Conclusions:**

We conclude that in typical Central-European grassland ecosystems, anthropogenic drivers rather decrease landscape-scale than local-scale biodiversity, because they tend to homogenize environmental conditions. The resulting species turn-over can stabilize local species richness but depletes the metacommunity, thereby posing future risks to the regional biodiversity.

**Supplementary Information:**

The online version contains supplementary material available at 10.1007/s10980-025-02256-0.

## Introduction

Biodiversity is globally declining, with habitat destruction and fragmentation via human land use as the most important drivers in terrestrial ecosystems, followed by over-exploitation, pollution and climate change (Díaz et al. 2019). Grasslands, in particular, belong to the most threatened (e.g., Jarvis et al. 2010; Millennium Ecosystem Assessment Program 2005) but also to the most species rich ecosystems worldwide, especially at local scales (Dengler et al. 2012; Wilson et al. 2012). In Central Europe, which is naturally dominated by various types of forest, the small patches of natural as well as the remnants of once widespread semi-natural grasslands contribute substantially to the regional meta-community and habitat diversity of the wider landscape (Habel et al. 2013; Sádlo et al. 2007; Veen and Aavik 2009). However, due to the loss of economic value and consequent decline of semi-natural grasslands over the past century, many former widespread and frequent species have faced strong population declines and are now listed on national and European Red Lists (Brummitt et al. 2015). As a result, natural and semi-natural grasslands rank high on both national and EU-wide conservation and restoration plans (Essl 2004; European Commission. Directorate General for the Environment and Ecosystems LTD., 2014).

While habitat loss and the consequent population decline of grassland specialists in Central Europe are undisputed, the development of local-scale plant diversity in remaining grasslands is less clear. In fact, meta-analyses have shown that worldwide trends of local-scale plant species richness over the last century do not follow a path of decline, but have remained stable in various types of habitats, including grasslands (Vellend et al. 2013, 2017). Although these results are still under debate (Primack et al. 2018), empirical data from central Europe largely point in the same direction (Jandt et al. 2022). The apparent contradiction between larger-scale loss and small-scale consistency of biodiversity points to linkages between local and regional-scale processes. In particular, it has been argued that a subset of rather generalist species is colonizing an increasing proportion of local habitats, while more specialized species are disappearing from local assemblages, and, eventually, from the larger-scale meta-community (Finderup Nielsen et al. 2019; Jandt et al. 2022; Thomas 2013). A homogenization of community composition, which should result from this supposed replacement of specialists by generalists, has been empirically demonstrated in several large-scale cross-habitat studies (Finderup Nielsen et al. 2019; Jandt et al. 2022; Prach and Kopecký, 2018). However, empirical case studies that directly test whether local stability is correlated with opposing population trends of different species groups at the landscape scale are still scarce (Eichenberg et al. 2021). Here, we undertake such a study using the Austrian national park Neusiedler See/Seewinkel as a model system.

In line with global trends, nutrient poor grasslands in Austrian lowlands have been facing severe habitat deterioration in the recent past (Grass et al. 2014; Hülber et al. 2017; Kapitany and Wrbka 2021). Hereby, the Neusiedler See/Seewinkel, located next to the Hungarian border in the Pannonian basin, represents one of the largest remaining extensively used grassland landscapes in eastern Austria. Shaped over centuries by free roaming herds of cattle, the Seewinkel still resembles steppic landscapes in continental Europe and provides a refuge for many grassland species within an IUCN national park (Zulka et al. 2022), including many Austrian Red-List species (Schratt-Ehrendorfer et al. 2022). Unique geomorphological conditions (Häusler and Frank 2007) foster an enrichment of upper soil layers with sodium carbonate and the formation of so called “sodic pans” which host highly endangered halophytes, making the regional grasslands even more unique (Albert et al. 2020). Nevertheless, pressures are manifold and the regional grassland area has decreased to a quarter of its 18^th^ century extent (Prinz et al. 2010). Presently, climate change (via increasing temperatures and drought periods) and agricultural groundwater withdrawal result in reduced water availability and salt concentrations in regional soils (Zimmermann-Timm and Teubner 2021). Eutrophication due to management intensification of surrounding agricultural land further threatens characteristic grassland species composition due to spill-over effects (Hülber et al. 2017).

In this study we use a unique dataset comprising 12 consecutive years of repeated plant community sampling to analyse the recent development of plant diversity of these extensive grasslands in the Seewinkel region in the light of global warming and agricultural intensification. Our research hereby links local species richness trends with landscape-scale incidence trends of individual species, aiming to determine whether a potential stability in local species richness results from opposing incidence trends—where some species are declining while others are increasing. More specifically, we try to answer the following research questions: (1) Are there any changes in the number of vascular plant species in local assemblages within the study period and is there an indication of homogenization? (2) How did the regional incidence of individual species across local assemblages change over time? (3) Are there opposing trends of regional incidence between initially abundant and initially rare species? (4) Can changes in species’ regional incidence be linked to their environmental preferences, especially their water, nutrient and thermal requirements, and are there any differences between species mentioned on national Red Lists and those that are not?

## Methods

### Study area

The study area “Seewinkel” is located in the Austrian federal state Burgenland between Lake Neusiedl and the Hungarian border at an average altitude of 115 m a.s.l. The area is situated in a transition zone between oceanic and continental climate with a mean annual temperature of 12 °C and 575 mm of annual precipitation. The landscape is dominated by viticulture and food crop production. Covering parts of Lake Neusiedl and adjacent land east of the lake in Austria and Hungary, the national park Neusiedler See—Seewinkel spans an area of 10.000 ha. The grasslands surveyed in this study are mostly situated in the buffer zone of the national park and can be divided into three major management types. Firstly, pastures are being grazed from April to October. The cattle are not fenced but range across the area, controlled by herdsmen, leading to a low grazing pressure. Secondly, meadows are mown once a year in late June including removal of mown material. The third type is fallow land (mostly former vineyards) which also gets mown once in early autumn to prevent shrub encroachment, but without removal of mown material.

### Study design and field surveys

**Fig. 1:**
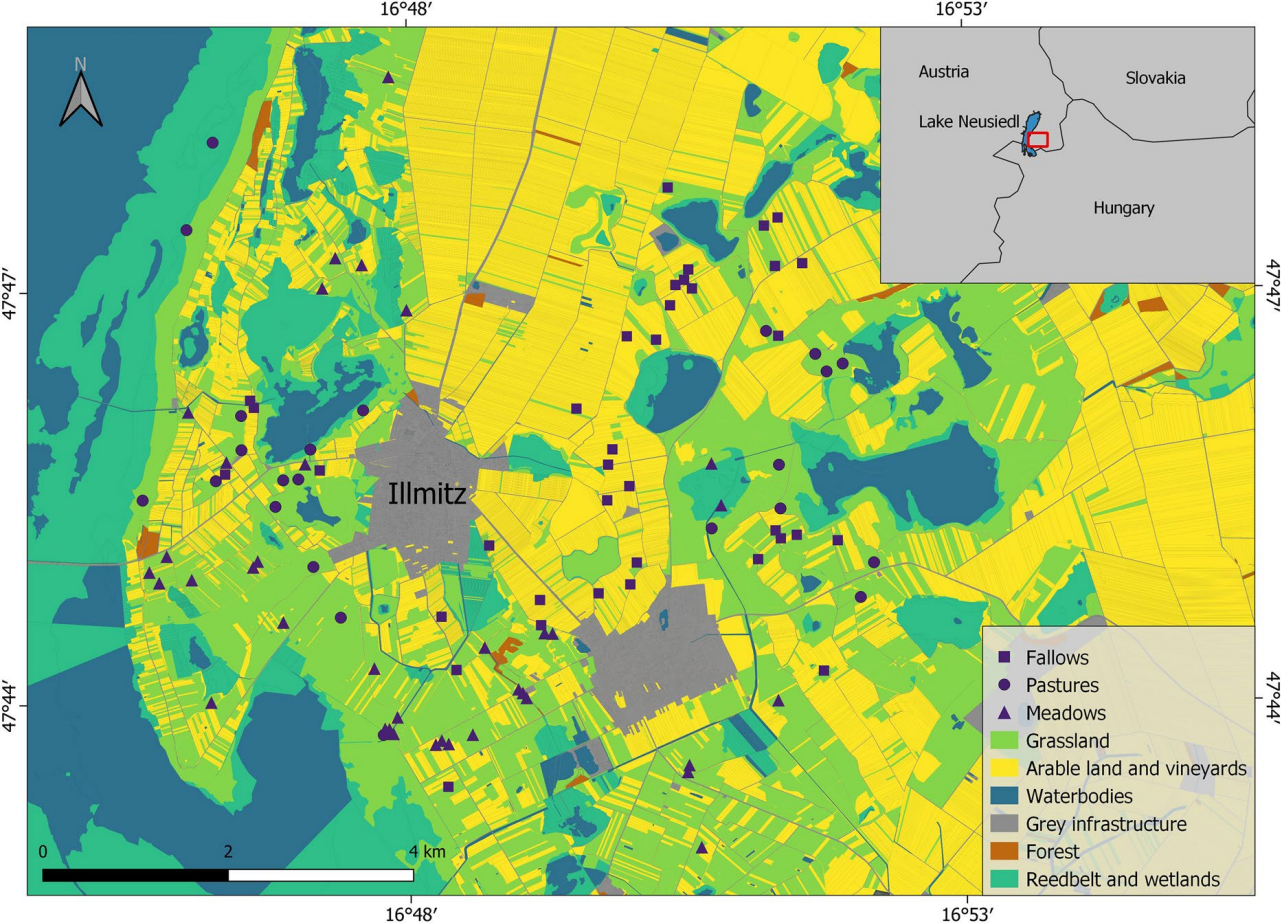
Location of the 102 surveyed sites within the study area

The grasslands were sampled annually in June from 2013 to 2024. Each year, we selected ten, or in some years slightly more, 50 × 50 m sites per management type (meadows, pastures and fallows) from a total pool of sites situated in a radius of 5 km around the village of Illmitz (Figure 1). A varying number of sites were resampled from year to year, due to occurring time-conflicts of sampling and mowing date as well as unforeseen management type changes on the sites; those not re-sampled were replaced by new ones. Over the 12 years this resulted in 102 sites sampled between 1 and 12 times - a table displaying all sampled sites for each year can be found in the supporting information. At each site, we selected four 2 × 5 m plots positioned in the corners of a transiently marked 30 × 30 m square, the center of which being relocated via GPS coordinates. For georeferencing we used Garmin Etrex10 devices (precision of ~3 m according to tests). For these plots, we collected a full list of all vascular plant species (determination followed Fischer et al. 2008), together with an estimate of their ground cover according to the 7-part Braun-Blanquet scale (Braun-Blanquet 1964).

### Data preparation and analysis

To remove possible inconsistencies in taxon determination among field botanists we aggregated all sub-species entries to the species level, and, in the case of some groups of species that are difficult to distinguish in the field, such as *Achillea millefolium*, to a species aggregate (*Achillea millefolium agg.*). Genus level determinations and species which were rare (= less than three records over the entire time series) and additionally not included in the regional checklist (Zulka et al. 2022) were excluded from species-level analyses but kept for the analysis of species richness trends, resulting in a total of more than 29.500 records within the study period. We also did not include (the few and rare) shrub species into the species level analyses, because we focused on the grassland species pool. The national threat status of species was taken from the Austrian Red-List of vascular plants (Schratt-Ehrendorfer et al. 2022). Furthermore, we characterized the environmental preferences of species by means of Ellenberg-type indicator values (EIV) taken from a comparative study of Tichy et al. (2023). Ecological indicator values are expert-based assessments of species’ realised niche optima along environmental gradients and have a long tradition in central European vegetation research for both characterizing species niches and inferring environmental conditions under which species assemblages grow (Ellenberg 1974; Landolt 2010).

We regressed the number of species per plot and the share of endangered species per plot, separately against time by means of generalized linear mixed models (GLMM, Brooks et al. 2017), assuming a negative binomial and a binomial distribution, respectively. To assess whether possibly changing local species composition indicates directional change of abiotic site conditions we calculated unweighted mean EIVs for each plot and, again, regressed these against time via linear mixed models, assuming a normal distribution and using the REML method (nlme-package, Pinheiro et al. 1999). We evaluated temporal trends of indicator values for temperature, moisture, nutrients and light, because we expected these to have changed most. In all of these models, we estimated a random intercept for the site ID and a random slope for the effect of year nested in site ID. Since the exact position of plots within a site varied from year to year, we refrained from adding a random effect for the plot ID. Additionally, we ran all the models for the three management types pasture, meadow and fallow separately. To visualize temporal changes in the species composition of the studied sites we performed non-metric multidimensional scaling (NMDS) based on Bray–Curtis dissimilarities, using the “vegan” R package (Oksanen et al. 2025). Due to within-site similarity of data points, we aggregated species entries from the plot to the site-level to ensure stability of the ordination. We calculated year-wise compositional centroids as the mean of site scores (NMDS axis 1 and 2) for all sites within each year to visualize between-year differences. We then calculated community-weighted means of EIVs (based on species cover) and fitted these to the NMDS. The resulting vectors indicate increasing or decreasing EIV values within the two-dimensional ordination. To evaluate possible homogenization, we tested for changes in the similarity of species composition between plots. We therefore calculated a mean Sørensen similarity index for each year (using the “forestmangr” package from Braga et al. 2018) and regressed the obtained values against time by means of ordinary least-squares regression (OLS). To test for changes in gamma diversity of the observed sites we regressed the total number of species identified per year against time, again using an OLS. To account for slight differences in the numbers of plots sampled per year, we weighted the number of species identified per year by the number of plots sampled per year. To analyse trends in the regional incidence (= frequency across plots) of individual species we calculated the proportion of plots occupied by a species per year (number of occurrences divided by the number of sampled plots) and regressed these proportions against time for each species separately by means of binomial generalized linear models using the logit link function. Subsequently, we tested for relationships between species’ temporal trends and environmental preferences by correlating the resulting species-wise regression coefficients to the species’ EIVs by means of OLS. We furthermore classified the 229 species into initially abundant, intermediate and rare species (i.e., occurring on average in more than 25%, 12.5 to 25% or less than 12.5% of the plots in the first three years) and tested for differences in the incidence trends of those groups. More specifically we evaluated whether initially abundant species had increased and rare ones decreased, in line with the basic hypothesis of stable community but declining meta-community diversity. We therefore conducted one-sided t tests for the three groups separately, comparing their incidence trends, i.e. the regression coefficients of their incidence across communities against time, against zero. For all species trend analyses, we only included species which were sampled at least once in at least 5 years within the study period. We used the R software (R Core Team 2023) for data analysis and crafting of graphs.

## Results

### Species richness and endangered species trends

**Fig. 2:**
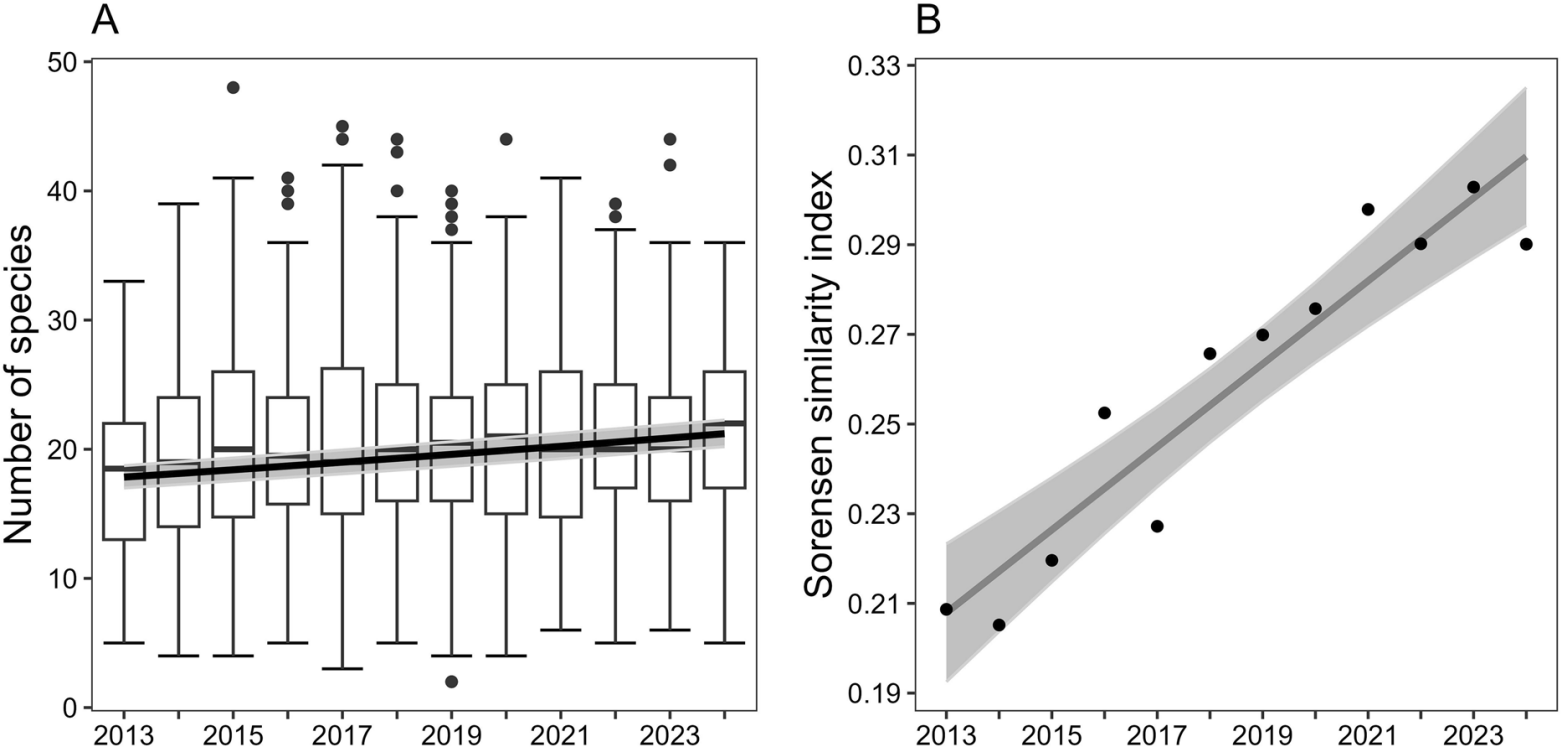
Changes in **A** mean number of vascular plant species per plot and **B** mean Sørensen similarity index between plots from 2013 to 2024 (black and dark grey lines, respectively show predicted values according to the OLS, light grey area shows 95% confidence intervals)

The regression model indicated an increase in mean local species richness from 18.3 species per plot (predicted values) in 2013 to 21 species in 2024 (z-test: p < 0.001, Figure 2A). These trends stayed significant when analyzing meadows and fallows separately—pastures showed no trend (see supporting information, Figure S1). However, regressing the overall number of species recorded per year over time did not show any significant results (see supporting information, Figure S2), indicating a possible homogenization of the plots. Indeed, the OLS confirmed a significant increase in similarity from on average 20.9% species overlap between plots in 2013 to 29% species overlap in 2024 (p < 0.001, Figure 2B). When regressing the number of endangered species per plot against time no significant trend could be found (data not shown).

### Ellenberg-type indicator values

**Fig. 3:**
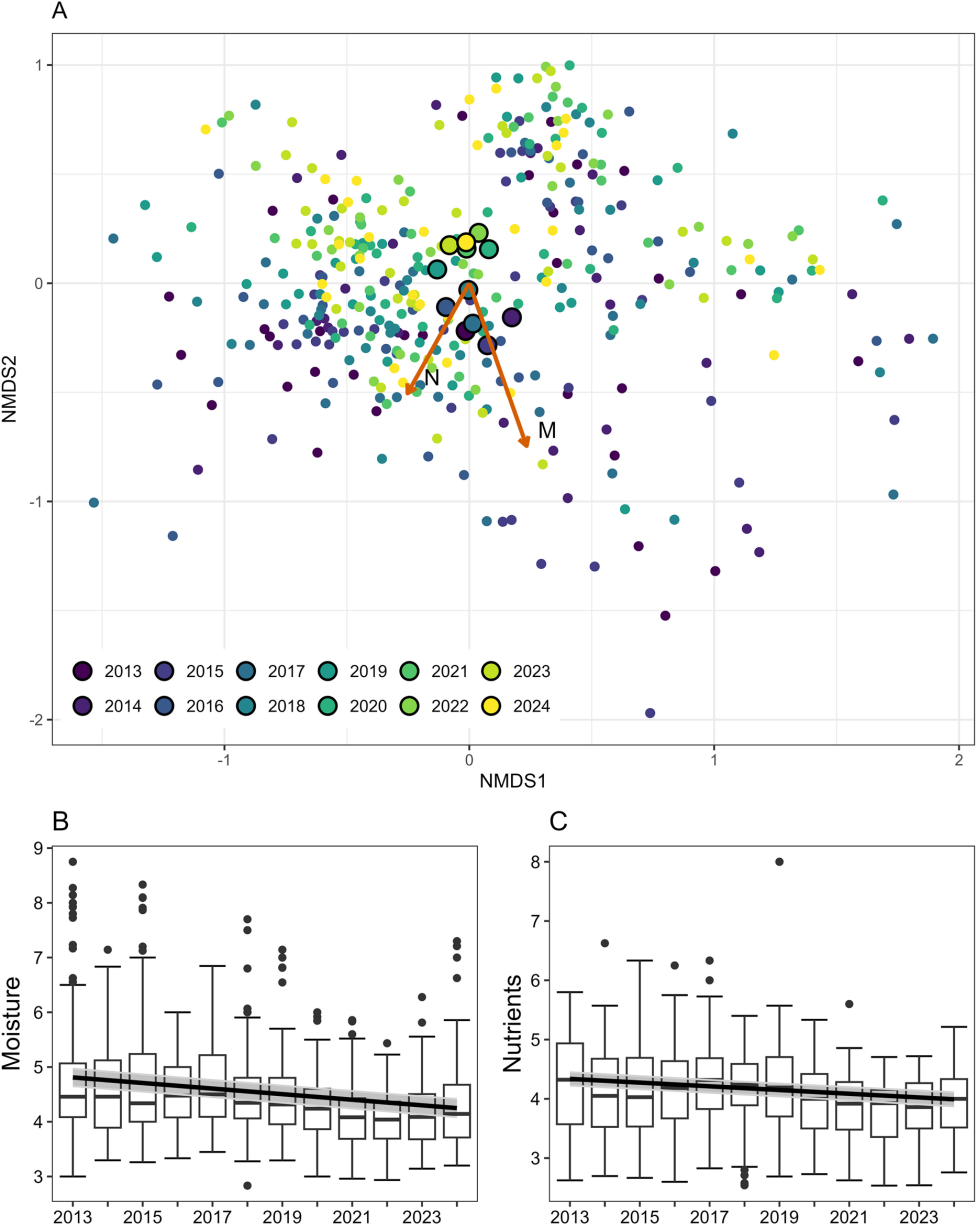
**A** NMDS results. Colors indicate yearly sampling points. Larger dots with black borders indicate yearly centroids. Orange arrows depict EIVs for moisture and nutrients fitted to the ordination. **B** Change in mean EIVs per plot from 2013 to 2024 for moisture and **C** nutrients. Trendlines depict predicted values of the linear mixed models. Adjacent grey area depicts 95% confidence intervals

Models indicated a decrease of mean EIVs for moisture and nutrients within the study period from 4.8 to 4.2 (predicted values) and from 4.3 to 4.0, respectively (t-test: p < 0.001, Figure 3A–C). Moisture trends were consistent across management types. For nutrients, pastures showed no significant trend when analysed separately. Mean EIVs for temperature and light slightly increased when analysing fallows separately, from 5.7 to 5.8 and from 7.5 to 7.6, respectively (p < 0.001, see supporting information, Figure S3), but trends were not significant when considering the whole data set (data not shown).

### Species trends

**Fig. 4:**
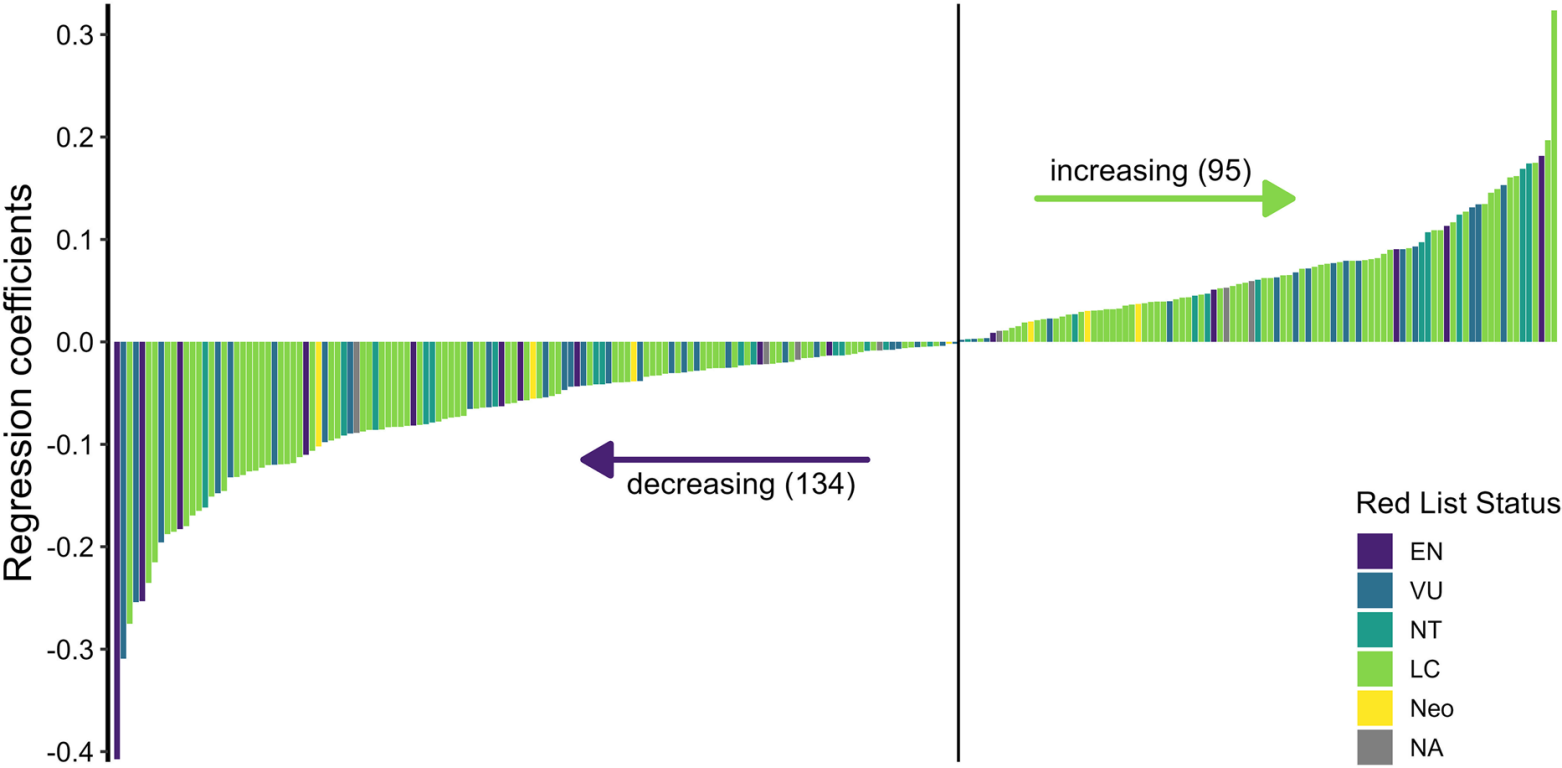
Species trends shown as regression coefficients on a log odds scale taken from binomial GLMs of 229 species. Bars are coloured according to Red List Status of the Austrian Red List (Ehrendorfer et al., 2022)

In total, 420 species were identified in the study period of which 229 occurred in at least 5 years. Out of these, 95 species (41.5%) increased in incidence per year whereas 134 (58.5%) decreased, resulting in a slight decrease on average across all 229 species (Figure 4). We found no significant differences between endangered and not endangered species in terms of temporal trends. The most pronounced “losers” and “winners” were, in the first case, *Scorzonera parviflora* (-10.1% decrease within the study period (predicted values)), *Scirpoides holoschoenus* (-7.7%) and *Phleum pratense* (-6.8%), and, in the second case, *Chenopodium album* (+8%), *Myosotis ramosissima* (+4.9%) and *Silene viscosa* (+4.5%).

**Fig. 5:**
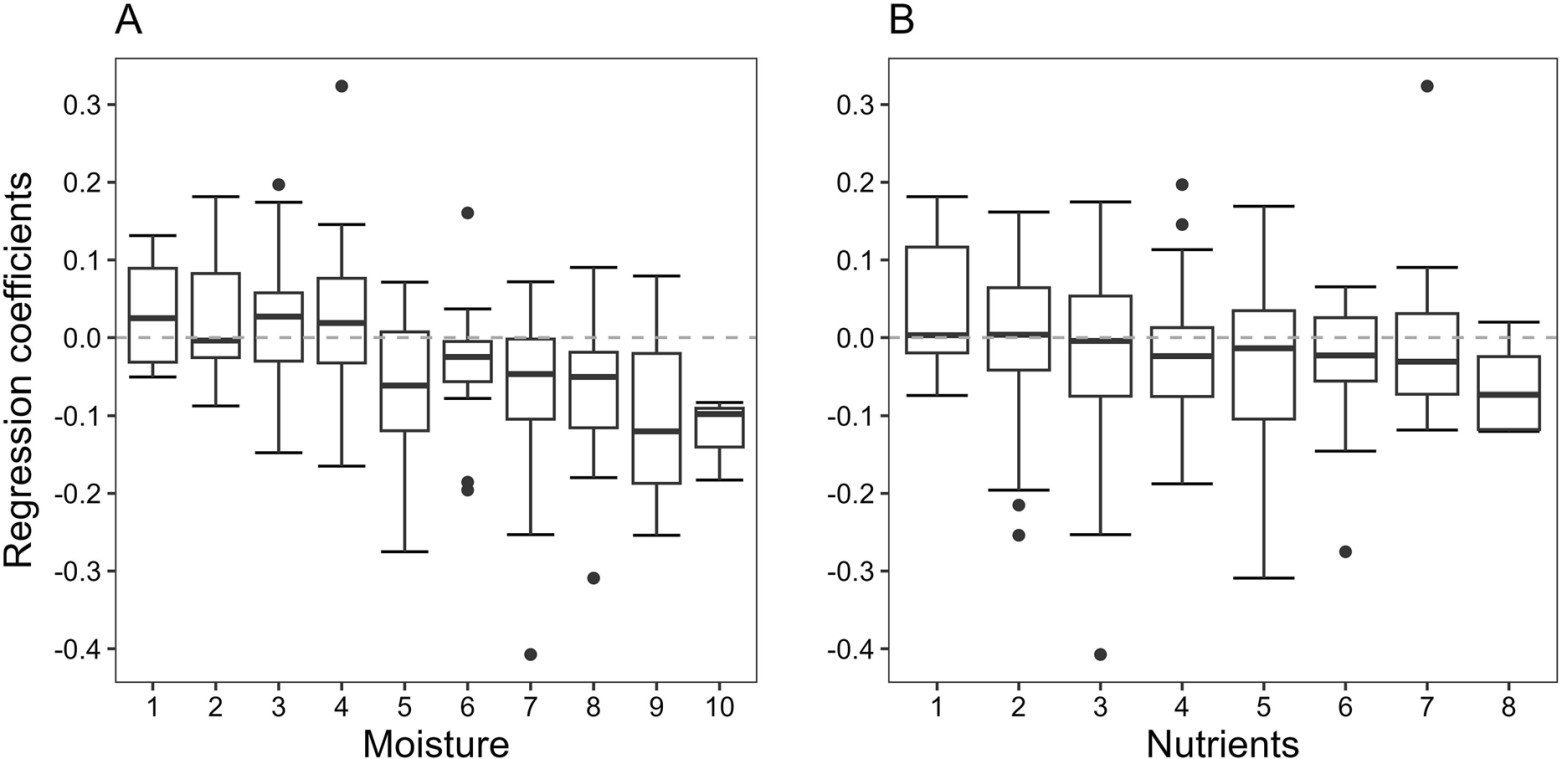
Mean regression coefficients (log odds scale—taken from binomial GLMs of 229 species) for each EIV class for **A** moisture and **B** nutrients

We found significant negative correlations between EIV for moisture and nutrients and species trends. The incidence of species adapted to drier conditions (moisture values 1-4, Figure 5A) tended to increase or stay the same (e.g., *Centaurea stoebe, Linum austriacum, Eryngium campestre,* EIV M 2, +11.5%, +11.6% and +10.7% increase, respectively) whereas species adapted to intermediate—moist conditions (values 5-8) showed overall decreasing trends. The decrease in incidence was even stronger for species adapted to wet conditions (values 9-10, e.g., *Schoenus nigricans* EIV M 9, -10.3% decrease). Species adapted to nutrient poor conditions (nutrient values 1-3, Figure 5B) tended to show no clear trend whereas species adapted to increasingly higher nutrient concentrations (values 4-8) decreased in incidence (e.g., *Artemisia vulgaris, Carduus acanthoides*, EIV N 8, -2.7% and -5.8% decrease, respectively). Trends for moisture were consistent when looking at the management types separately whereas for nutrients, trends remained significant only when looking at fallows separately (see supporting information, Figure S4). EIVs for temperature and light showed no significant correlations between species trends and EIVs.

**Fig. 6:**
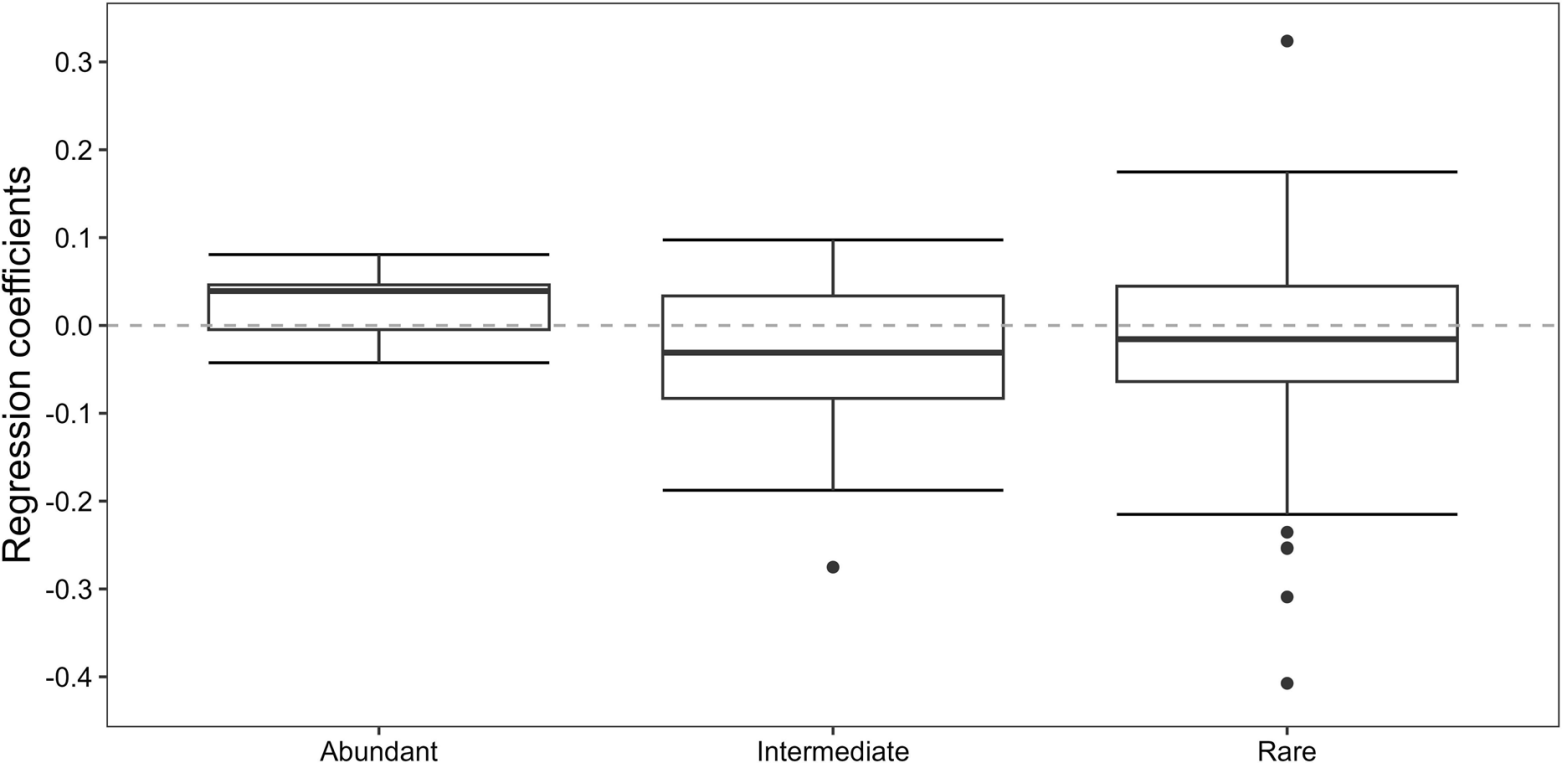
Comparison between mean regression coefficients (log odds scale—taken from binomial GLMs of 229 species) for initially **a** abundant (n =13), **b** intermediate (n = 31) and **c** rare species (n = 185)

Finally, we compared incidence trends between initially abundant, intermediate and rare species. While incidence trends were significantly positive for initially abundant species (mean value = 0.025, t = 2.4, df = 12, p < 0.05, Figure 6), they were significantly negative for intermediate and rare species (mean value = -0.032 and -0.014, t = -2.1 and -2, df = 30 and 184, p < 0.05).

## Discussion

### General trends

Despite the relatively short period of 12 years our study identified significant changes in the plant community of Seewinkel grasslands. The richness of local assemblages has increased from a mean of 18.3 species per plot in 2013 to 21 species in 2024. However, this increase in local diversity was neither reflected in regional diversity trends, i.e., the total number of species recorded per year did not change, nor in individual species trends which slightly declined, on average. Instead, we found a significant homogenization of local assemblages from on average 21% overlapping species between any two plots in 2013 to 29% overlapping species in 2024. This homogenization was driven by opposing trends of initially abundant and less abundant species, with the former becoming even more frequent while the incidence of the latter decreased. In addition, indicator value analyses suggest an ongoing turn-over from species preferring wet or moist to species preferring drier conditions. Finally, both the number of endangered species per plot and individual trends of endangered species showed no significant changes within the study period.

The opposing trends between plot level diversity and individual species incidence underline the notion that local species richness trends alone must be treated with caution when used for evaluating biodiversity trends or the effectiveness of conservation measures. Our findings are in line with scattered local (Schwaiger et al. 2022) and some larger scales studies from Germany (Jandt et al. 2022) and Denmark (Finderup Nielsen et al. 2019) which showed that general trends of a stable local and regional species richness were opposed by overall declining trends of individual species occurrence. Looking at the different levels of diversity, our findings translate to an increase in alpha, a decrease in beta and a constant gamma diversity. Such a combination might precede larger scale biodiversity loss, where a few ubiquitous generalists steadily replace less common specialists (Sweeney and Jarzyna 2022), ultimately leading to a decline in gamma diversity. Monitoring biodiversity trends must hence go beyond local species richness and include individual species’ trends at larger scales to detect degradation not evident at the plot scale.

### Changes in environmental conditions (Ellenberg-type indicator values)

We inferred changes in abiotic site conditions from the analysis of Ellenberg indicator values. Here, results were mostly in line when comparing the plot level analysis and individual species trends. As expected, mean moisture values on the plots decreased, a trend that was consistent across management types and points to reduced water availability in many parts of the study landscape. By correlating species trends with EIV classes we could draw an even clearer picture. While species adapted to dry conditions showed clear increases within the study period, species adapted to intermediate conditions showed no, or slightly decreasing trends, and species adapted to wet conditions showed strong decreasing trends. A plausible hypothesis is that these temporal changes are driven by climatic trends, especially as some of the later years such as 2018–2020 were characterized by very dry summers in central Europe. However, an additional analysis conducted to evaluate this assumption did not provide support for it. While the regional mean yearly SPEI (standardized precipitation evapotranspiration index; Haslinger and Bartsch 2016) correlated with EIV for moisture (i.e., moisture values were higher in wetter years and vice versa; see supporting information) we found, contrasting to EIV, no significant temporal trend of SPEI. We conclude that annual weather fluctuations had an impact on annual EIV for moisture, perhaps mostly via the incidence of annuals, but that the found temporal trend in EIV was not driven by a trend in SPEI. Trends for nutrient values were not in line with our expectations. Nutrient values on the plots decreased, as did incidences of species with a preference for high soil nutrient contents, indicating reduced nutrient levels in soils. This contrasts with general trends in grasslands (e.g., Hülber et al. 2017; Schwaiger et al. 2022) and can probably not be explained via decreasing soil moisture contents as prior studies have disproved a negative effect of the latter on soil nutrient availability in grasslands (Hartmann and Niklaus 2012). However, only fallows showed a significant decrease in nutrient values and are hence the main driver of this trend. Therefore, these results can be interpreted as an effect of the management of fallows in the region (one cut in late summer to avoid shrub encroachment). As fallow arable land usually exhibits high soil nutrient levels after the cessation of use (Aerts et al. 1995) a decrease of nutrient levels via extensive management is likely. Nevertheless, also for meadows and pastures no eutrophication could be detected within the study period, a fact which points to the effectivity of extensive management (one cut in early summer and less than 1 LFU/ha, respectively) of grasslands in the region. For fallows only, we could furthermore detect slight but significant increases in mean light and temperature values on the plot level. This indicates that the vegetation of those sites which is usually dominated by tall growing forbs in an initial state (Marrs 1993) becomes more open as typical fallow species decrease in abundance and species of open habitats with higher light and temperature demands increase. Again, this can probably be explained by the extensive management of the sites directing the development towards more meadow-like plant communities (Kardol et al. 2008).

### Agricultural management

Our trends show that the extensive management of grasslands in the Seewinkel region is suitable to avoid signs of eutrophication, at least within the study period. Furthermore, the management of fallow vineyards seems to have a positive effect as those sites showed decreasing nutrient levels and a development towards more meadow-like species compositions. What seems to be a threat to the current species composition of regional grasslands is the ongoing desiccation of regional soils. On the one hand, climate change drives temperature rise and precipitation patterns become more variable, leading to increased periods of drought and higher evapotranspiration. On the other hand, regional agriculture lowers groundwater levels due to the cultivation of irrigation-intensive crops such as potatoes, a practice that has increased strongly in recent decades. Water loss from agriculture is additionally intensified by climate change as irrigation demand rises with drier conditions. As our data demonstrate, this development had a clear impact on the regional flora within a comparatively short period of only 12 years. Given that the vegetation of wetlands and salt marshes belongs to the most valuable components of the regional, and even national, diversity regulations on irrigation measures appear key to prevent a further degradation of these habitats.

The relatively short monitoring period and varying sampling frequencies among sites (see supporting information) are key limitations of this study. However, the use of incidence-based trend analyses likely reduces susceptibility to inconsistent sampling effort, thereby supporting the robustness of the main findings. Future monitoring should prioritize consistent, repeated sampling of a fixed set of sites to extend temporal coverage and enable a more detailed assessment of site-specific dynamics.

### Conclusion

By linking local species richness and regional species incidence trends our study clearly supports the hypothesis that stable or even increasing local plant species richness is an insufficient means of monitoring conservation-relevant biodiversity trends. In the study area, stable local richness masks an ongoing loss of rarer species from the metacommunity, accompanied by a declining beta diversity. By analyzing species environmental preferences, we found evidence that species in need of higher soil water contents are decreasing in the area and being replaced by species adapted to drier conditions. In the long run these co-occurring processes may or may not result in an overall decline of the regional vascular plant diversity. Most certainly though, these processes will change the character of the regional grasslands and lead to the loss of at least part of the biota that the national park is meant to protect.

Analyzing biodiversity changes on intermediate scales might close a knowledge gap between supra-regional and plot-scale diversity trends. While supra-regional studies point to large-scale phenomena, mostly without identifying accomplishable pathways of action, plot-scale analyses are often subject to small-scale stochastic processes and can only inform about site-level management. In contrast, regional scale studies can identify trends and drivers on an intermediate level while still taking local-scale processes into account, which allows to infer regionally applicable management guidelines. We therefore stress the importance of conducting studies that connect local and landscape-to-regional processes in plant community changes and identify possible drivers which in turn may allow to counteract ongoing diversity trends.

## Supplementary Information

Below is the link to the electronic supplementary material.

Supplementary file1 (XLSX 14 KB)

Supplementary file2 (XLSX 24 KB)

Supplementary file3 (XLSX 1942 KB)

Supplementary file4 (DOCX 642 KB)

Supplementary file4 (TXT 48 KB)

## Data Availability

All original data supporting the findings of this study are available within the paper and its Supplementary Information. All third party data used for the analysis are cited in the paper and publicly available.
