# Supplementary material for: Local-scale increase masks landscape-scale loss of species richness in managed Pannonian grasslands
Source: Landsc Ecol. 2025 Nov 25;41(1):10. doi: 10.1007/s10980-025-02256-0 (PMC12738669; doi:10.1007/s10980-025-02256-0)
Supplement: Supplementary file 4 — Supplementary file4 (DOCX 642 KB) [file 10980_2025_2256_MOESM4_ESM.docx]

Supporting information


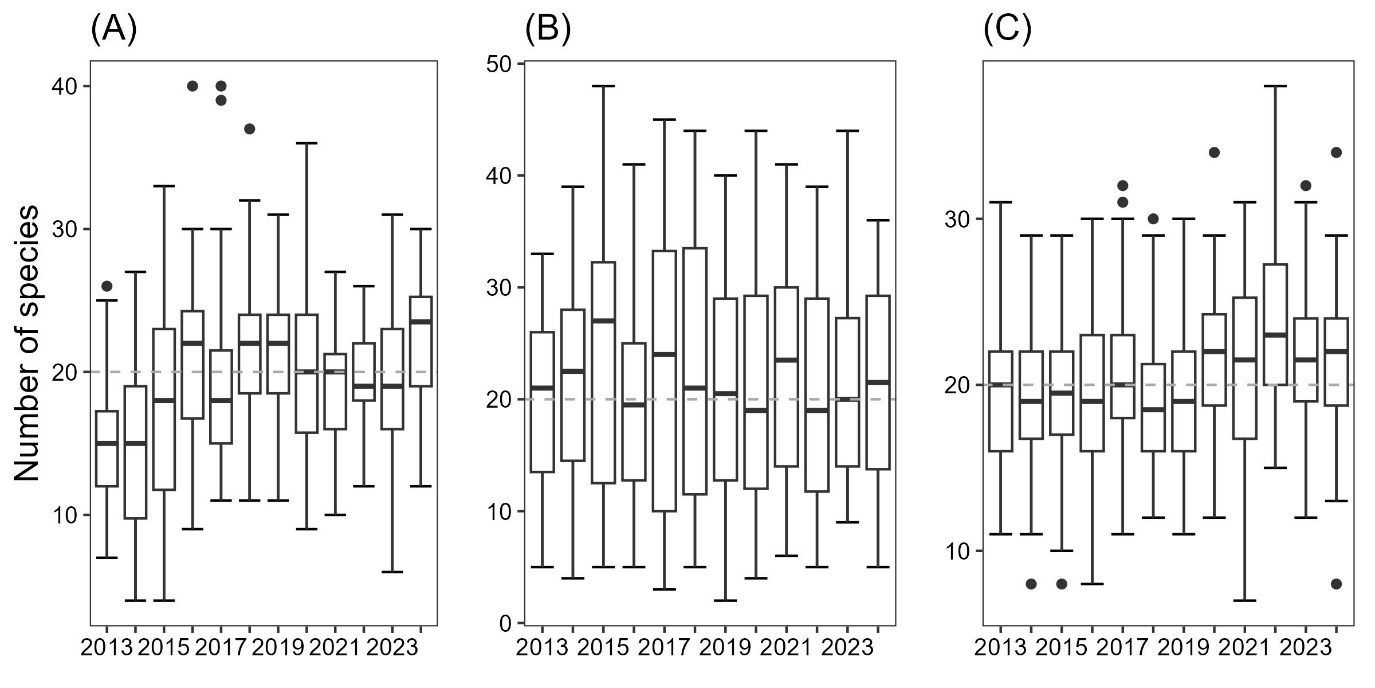


Figure S1/ Changes in mean number of vascular plant species per plot between 2013 and 2024 for A) meadows, B) pastures and C) fallows


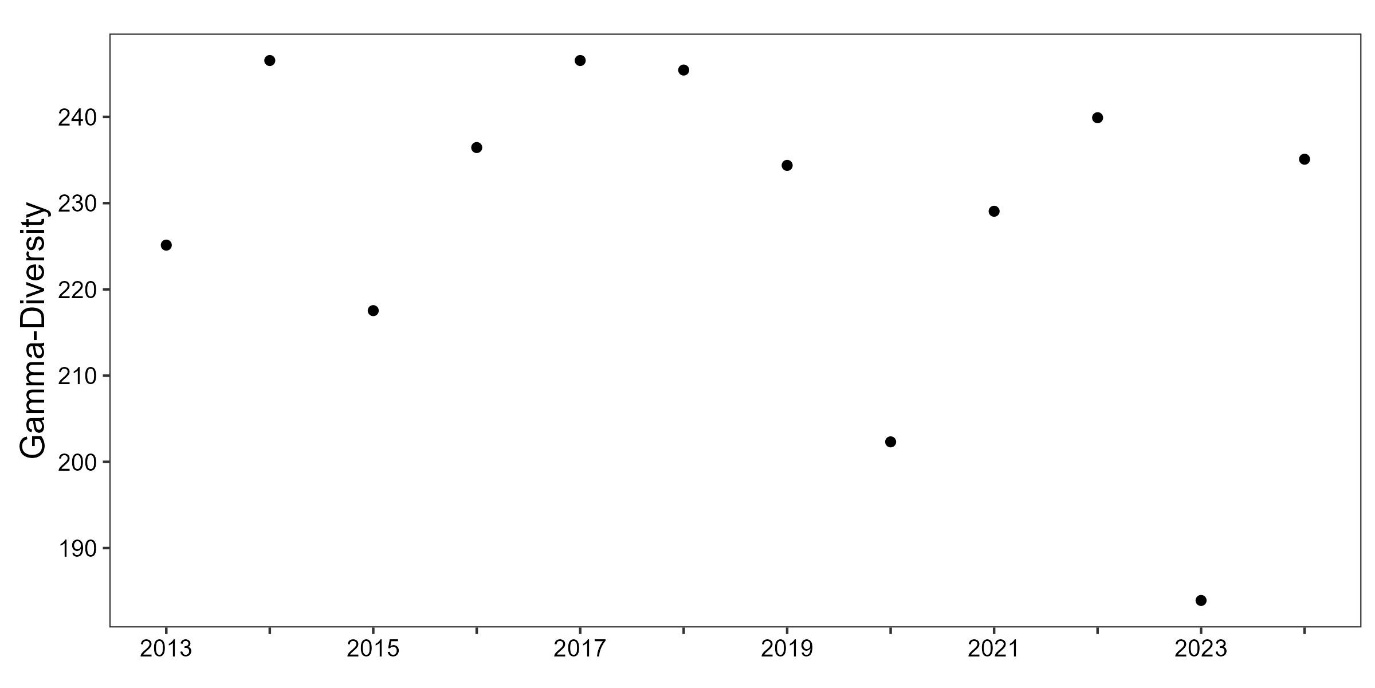


Figure S2/ Weighted gamma diversity (shown as total number of species identified per year) from 2013 to 2024. No significant temporal trend could be found.


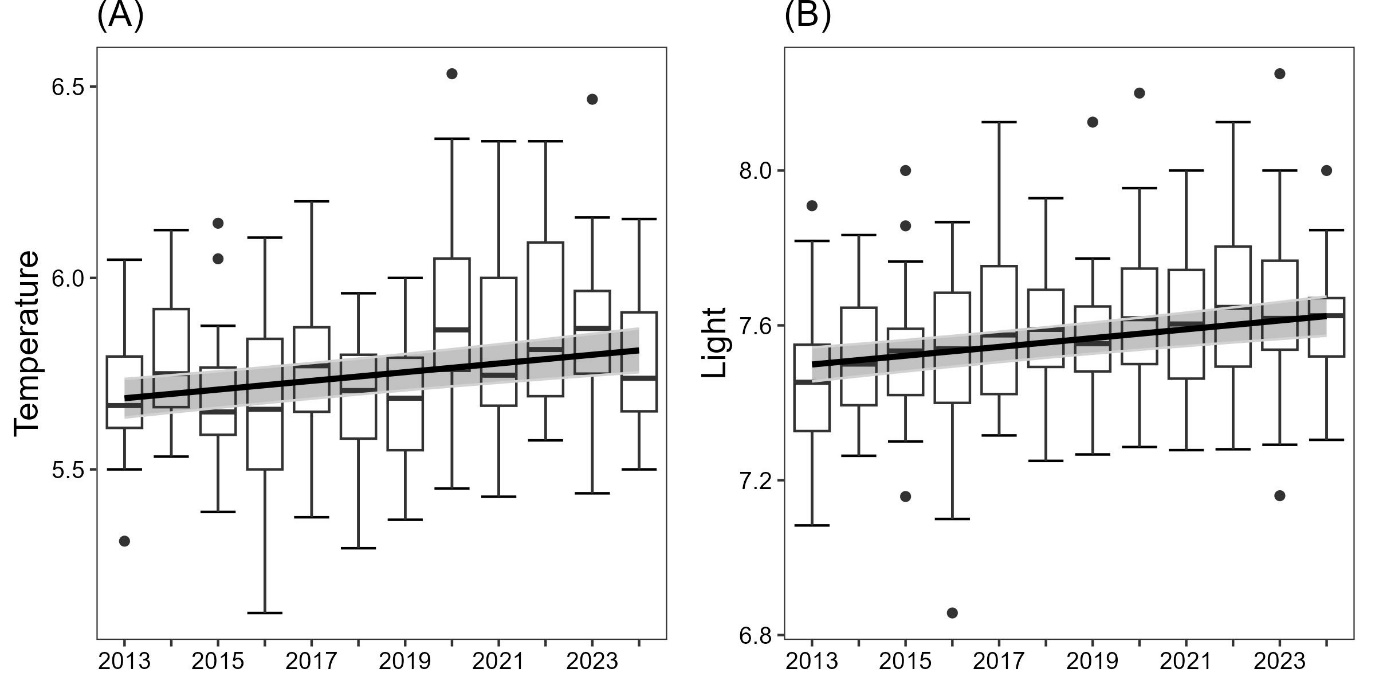


Figure S3/ Change in mean EIVs per plot from 2013 to 2024 for a) temperature and b) light for fallows only. Colored trendlines depict predicted values of the linear mixed models. Adjacent grey area depicts 95% confidence intervals.


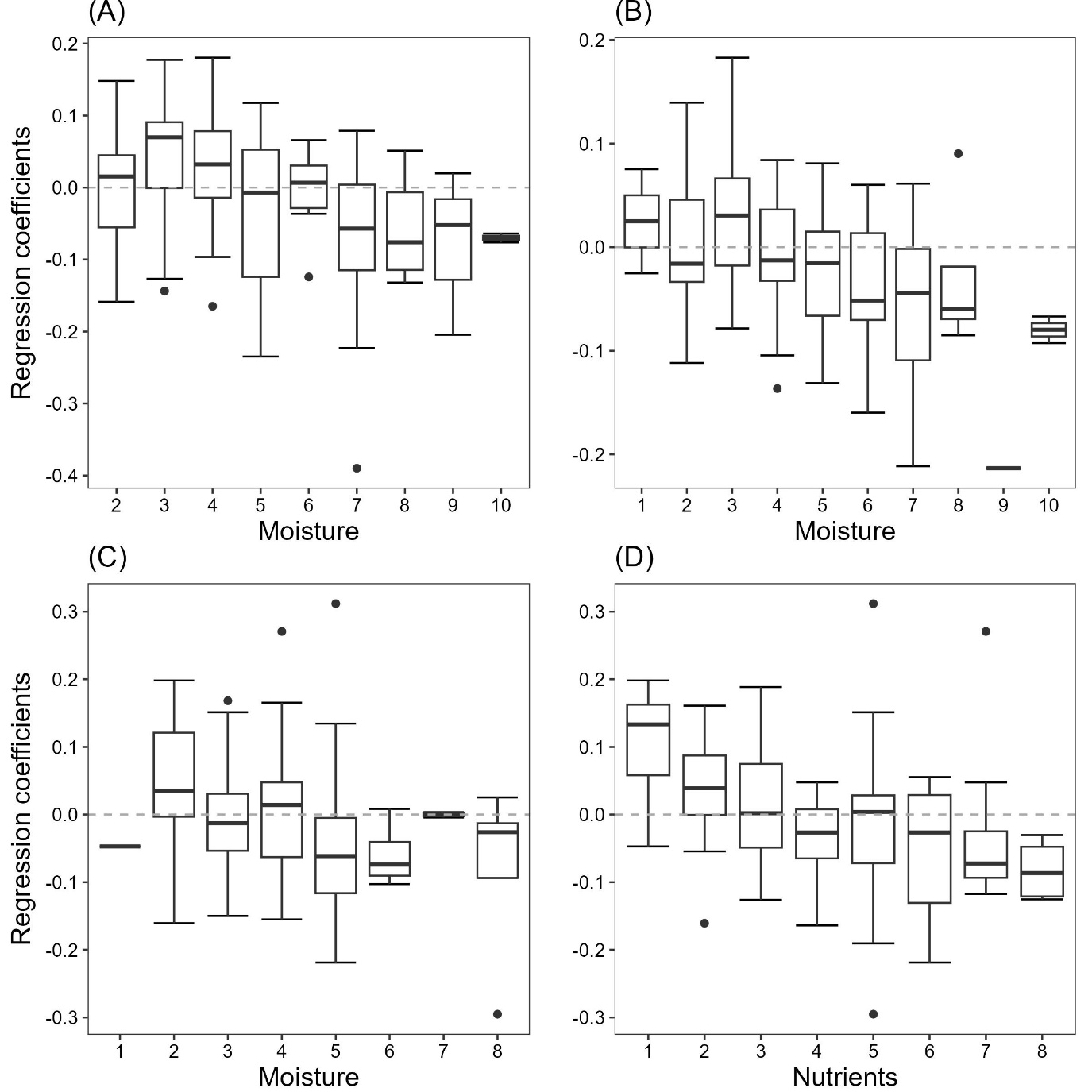


Figure S4/ Mean regression coefficients (log odds scale - taken from binomial GLMs of 229 species) for each EIV class for moisture, depicted separately for A) meadows, B) pastures and C) fallows and D) mean regression coefficients for each EIV class for nutrients depicted for fallows

**SPEI vs. EIV moisture values**

To test for correlations between mean EIV moisture values and yearly mean SPEI (standardized precipitation evapotranspiration index) we regressed the former against the latter via a linear mixed model, assuming a normal distribution and using the REML method. We estimated a random intercept for the site ID and a random slope for the effect of year nested in site ID. SPEI values for Austria are available as open data via <https://data.hub.geosphere.at> in a resolution of 1km² with daily data points. We selected all data points spatially overlapping with our study area and calculated a mean yearly value.

The model indicated a significant positive effect of mean yearly SPEI on mean EIV values for moisture (β = 0.26 ± 0.07 SE, t(1354) = 3.55, p < 0.001).

We furthermore regressed mean yearly SPEI against time by means of ordinary least-squares regression (OLS) to check for temporal trends. The OLS could not confirm a significant trend of SPEI over the study period (Figure S4).


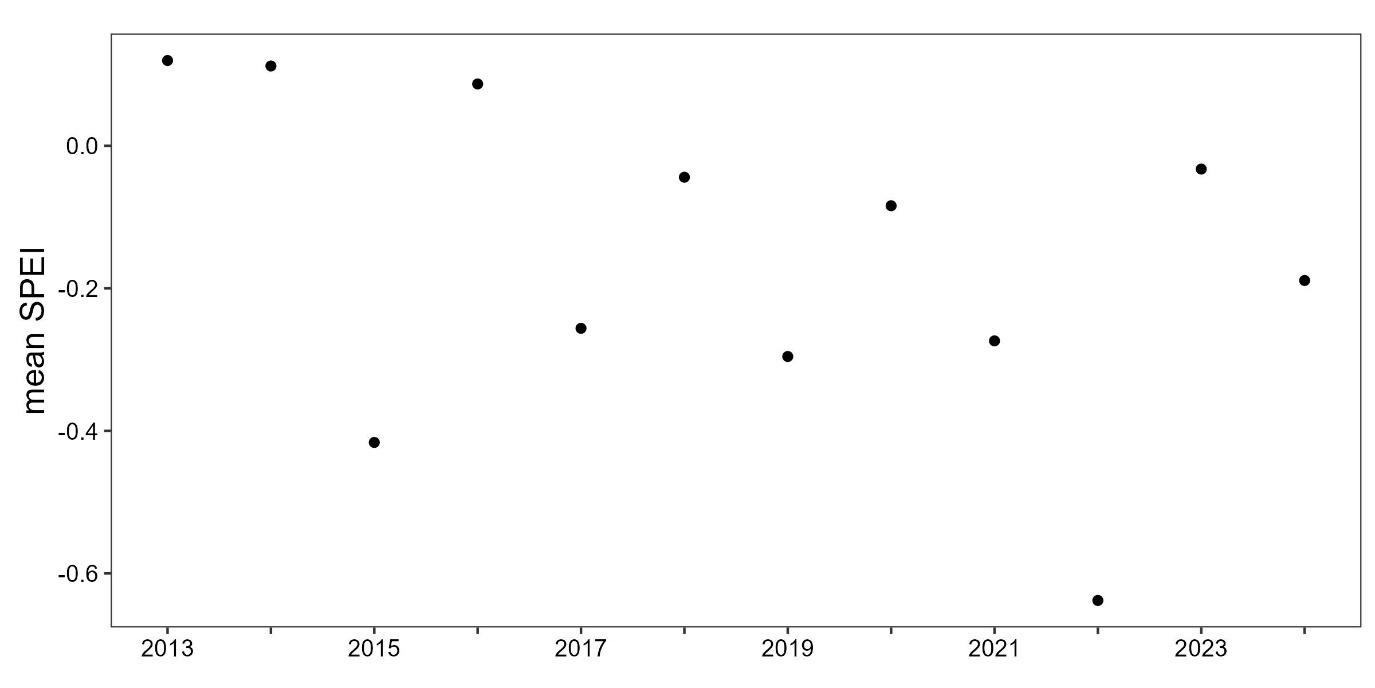


Figure S5/ Temporal trend of mean SPEI (standardized precipitation evapotranspiration index) over the study period. Values above zero indicate higher water availability compared to long-term trends. Values below zero indicate less water availablity compared to long-term trends. SPEI values derived from data.hub.geosphere.at

Table 1/ Full record of sampled sites (n =102)

| **Site_ ID** | **2013** | **2014** | **2015** | **2016** | **2017** | **2018** | **2019** | **2020** | **2021** | **2022** | **2023** | **2024** |
| --- | --- | --- | --- | --- | --- | --- | --- | --- | --- | --- | --- | --- |
| B01 |  | x |  | x | x |  |  |  |  |  | x | x |
| B03 | x |  | x | x | x | x | x | x | x | x | x | x |
| B06 |  |  | x |  |  |  |  |  |  |  |  |  |
| B07 | x |  |  | x |  |  |  |  |  |  |  |  |
| B08 |  |  |  | x |  |  |  |  |  |  |  |  |
| B09 |  |  |  | x |  |  |  |  |  |  |  |  |
| B10 |  | x | x |  | x | x | x | x | x | x | x | x |
| B11 |  |  | x |  |  |  |  |  |  |  |  |  |
| B12 | x | x |  |  |  |  |  |  |  |  |  |  |
| B13 | x |  |  |  |  |  |  |  |  |  |  |  |
| B14 |  | x |  |  |  |  |  |  |  |  |  |  |
| B16 |  | x |  |  |  |  |  |  |  |  | x |  |
| B17 |  |  | x |  |  | x | x | x | x | x | x | x |
| B19 |  |  |  | x |  |  |  |  |  |  |  |  |
| B20 | x |  |  | x |  |  |  |  |  |  |  |  |
| B21 | x | x |  |  |  |  |  |  |  |  | x | x |
| B23 | x |  |  |  |  |  |  |  |  |  |  |  |
| B24 | x |  |  |  |  |  |  |  |  |  |  |  |
| B25 |  | x |  |  |  |  |  | x | x | x |  | x |
| B26 |  |  | x |  |  | x | x |  |  |  |  |  |
| B28 |  |  |  |  | x |  |  |  |  |  |  |  |
| B29 |  |  |  |  | x | x | x |  |  |  |  |  |
| B30 |  |  |  | x |  |  |  |  |  |  |  |  |
| B31 |  | x | x | x | x |  |  |  |  |  | x | x |
| B32 |  |  |  |  | x |  |  |  |  |  |  |  |
| B33 |  |  |  |  |  | x | x |  |  |  |  |  |
| B34 |  | x |  |  |  |  |  |  |  |  |  |  |
| B35 |  |  |  |  |  | x | x | x | x | x | x |  |
| B36 |  |  |  |  | x | x | x |  |  |  |  |  |
| B37 |  |  | x |  | x | x | x |  |  |  | x | x |
| B40 | x |  |  | x |  | x | x | x | x | x | x | x |
| B41 | x |  |  |  |  |  |  |  |  |  |  |  |
| B42 |  |  | x |  | x |  |  | x |  |  | x | x |
| B50 | x |  |  |  |  |  |  |  |  |  |  |  |
| B53 |  |  |  |  |  |  |  | x | x | x |  |  |
| B55 |  |  |  |  |  |  |  | x |  |  | x |  |
| B57 |  |  |  |  |  |  |  | x | x | x | x |  |
| B60 |  |  | x |  |  |  |  |  |  |  |  |  |
| B70 |  |  | x |  |  |  |  |  |  |  |  |  |
| B75 |  |  |  | x |  |  |  |  |  |  |  |  |
| H04 | x | x |  |  |  |  |  |  |  |  |  |  |
| H16 |  |  |  |  |  |  |  | x |  |  |  |  |
| H18 |  | x | x | x | x | x | x | x | x | x | x | x |
| H19 |  | x |  |  |  |  |  | x |  |  | x | x |
| H20 |  | x |  |  |  |  |  |  |  |  |  |  |
| H22 | x | x | x | x | x | x | x | x | x | x | x | x |
| H23 |  |  | x |  | x | x | x | x | x | x |  | x |
| H24 | x |  |  | x |  |  |  |  |  |  |  |  |
| H26 |  |  | x | x | x | x | x | x |  |  | x | x |
| H27 |  | x | x | x | x | x | x | x | x | x | x | x |
| H29 |  | x | x | x | x | x | x | x | x | x | x | x |
| H31 | x |  |  |  |  |  |  |  |  |  |  |  |
| H32 | x | x | x | x | x | x | x | x | x | x | x | x |
| H34 | x |  |  | x |  | x | x |  |  |  |  | x |
| H36 |  |  | x |  | x | x | x |  |  |  | x | x |
| H38 | x | x | x | x | x |  |  | x | x | x | x | x |
| H40 | x | x | x | x | x | x | x | x | x | x | x | x |
| H42 | x |  |  |  |  |  |  |  |  |  |  |  |
| H44 | x |  |  |  |  |  |  |  |  |  |  |  |
| H50 | x |  | x |  |  |  |  |  |  |  |  |  |
| H51 | x |  |  |  |  |  |  |  |  |  |  |  |
| H52 |  |  |  |  |  |  |  | x |  |  | x |  |
| H56 |  |  |  |  |  |  |  | x | x | x |  |  |
| H119 |  |  |  |  |  |  |  | x | x | x | x |  |
| W01 |  | x | x |  |  |  |  |  |  |  |  |  |
| W05 |  | x | x |  |  |  |  |  |  |  | x | x |
| W06 | x |  | x |  |  |  |  |  |  |  |  |  |
| W11 |  |  | x | x | x |  |  |  |  |  | x |  |
| W12 |  |  | x |  |  |  |  |  |  |  |  |  |
| W13 |  | x | x |  | x |  |  | x | x | x | x | x |
| W14 |  | x | x | x |  | x | x |  |  |  | x | x |
| W15 | x |  |  |  |  |  |  |  |  |  |  |  |
| W16 |  | x | x |  |  | x | x |  |  |  | x | x |
| W17 | x |  |  |  |  |  |  |  |  |  |  |  |
| W18 |  | x |  |  |  |  |  |  |  |  | x |  |
| W19 | x |  |  |  |  |  |  |  |  |  |  |  |
| W20 | x |  |  |  |  |  |  |  |  |  |  |  |
| W27 |  |  |  |  |  |  |  |  |  |  |  | x |
| W41 |  |  |  |  |  |  |  | x | x | x | x |  |
| W43 | x |  |  | x | x |  |  | x | x | x | x | x |
| W45 | x |  |  |  |  |  |  |  |  |  |  |  |
| W47 |  |  |  | x | x | x | x | x | x | x | x |  |
| W48 |  |  |  |  | x |  |  |  |  |  |  |  |
| W49 |  |  |  | x | x |  |  | x | x | x |  |  |
| W50 | x |  |  |  |  | x | x |  |  |  |  | x |
| W51 |  | x |  | x | x | x | x | x |  |  | x | x |
| W52 |  |  |  | x |  |  |  | x | x | x |  |  |
| W53 |  |  |  |  |  | x | x |  |  |  |  |  |
| W56 |  |  |  |  | x |  |  |  |  |  |  |  |
| W57 |  |  |  |  |  | x | x |  |  |  |  |  |
| W58 |  |  |  |  |  | x | x |  |  |  |  |  |
| W59 |  |  |  |  |  |  |  | x | x | x | x |  |
| W60 |  |  |  |  | x |  |  |  |  |  |  |  |
| W61 |  |  |  |  |  | x | x |  |  |  |  |  |
| W62 |  |  |  | x |  |  |  |  |  |  |  |  |
| W63 |  |  |  | x |  | x | x |  |  |  |  |  |
| W64 |  |  |  |  | x |  |  |  |  |  |  |  |
| W65 |  |  |  |  |  |  |  | x | x | x | x |  |
| W70 |  |  | x |  |  |  |  |  |  |  |  |  |
| W80 |  |  | x |  |  |  |  |  |  |  |  |  |
| W81 |  |  |  |  |  |  |  | x |  |  | x |  |
| W84 |  |  |  |  |  |  |  | x | x |  |  |  |
